# Nebulization of low-dose aspirin ameliorates Huntington’s pathology in N171-82Q transgenic mice

**DOI:** 10.1515/nipt-2023-0026

**Published:** 2024-02-09

**Authors:** Susanta Mondal, Shelby Prieto, Suresh B. Rangasamy, Debashis Dutta, Kalipada Pahan

**Affiliations:** Department of Neurological Sciences, Rush University Medical Center, Chicago, USA; Division of Research and Development, Jesse Brown Veterans Affairs Medical Center, Chicago, USA

**Keywords:** Huntington’s disease, aspirin, huntingtin pathology, glial inflammation, neuroprotection

## Abstract

Huntington Disease (HD), a devastating hereditary neurodegenerative disorder, is caused by expanded CAG trinucleotide repeats in the huntingtin gene (*Htt*) on chromosome 4. Currently, there is no effective therapy for HD. Although aspirin, acetylsalicylic acid, is one of the most widely-used analgesics throughout the world, it has some side effects. Even at low doses, oral aspirin can cause gastrointestinal symptoms, such as heartburn, upset stomach, or pain. Therefore, to bypass the direct exposure of aspirin to stomach, here, we described a new mode of use of aspirin and demonstrated that nebulization of low-dose of aspirin (10 μg/mouse/d=0.4 mg/kg body wt/d roughly equivalent to 28 mg/adult human/d) alleviated HD pathology in N171-82Q transgenic mice. Our immunohistochemical and western blot studies showed that daily aspirin nebulization significantly reduced glial activation, inflammation and huntingtin pathology in striatum and cortex of N171-82Q mice. Aspirin nebulization also protected transgenic mice from brain volume shrinkage and improved general motor behaviors. Collectively, these results highlight that nebulization of low-dose aspirin may have therapeutic potential in the treatment of HD.

## Introduction

Huntington’s disease (HD) is a rare inherited devastating neurodegenerative disorder characterized by a triad of motor, cognitive and psychiatric features. HD is caused by CAG trinucleotide repeat expansion in the *huntingtin* (*Htt*) gene, which translates into Htt protein with an expanded polyglutamine tract (polyQ) that is prone to misfolding. The prevalence of HD is 4–10 per 100,000 in the western population and the mean age of onset is 40 years [1]. The expanded CAG repeat produces a dysfunctional, unfolded, and aggregated Htt protein, called mutated Htt (mHtt) [2]. Aggregates expand through the interactions between the polyQ repeat regions, which adopt a β-sheet conformations favoring the aggregation of Htt in the neuron [3]. Diverse cellular pathways are disturbed by direct or indirect interference of soluble, oligomeric and/or aggregated Htt such as transcriptional dysregulation of basal and inducible gene expression, impaired protein degradation, altered protein folding, and disrupted synaptic signaling.

Although pathogenic Htt is ubiquitously expressed in different kind of neural cells in the brain, neurodegeneration in HD is highly selective for striatal GABAergic medium‐sized spiny neurons (MSNs) that project to the substantia nigra (SN) and globus pallidus [4]. These neurons are the first to die in early-stage HD, and they die in the greatest numbers compared to other neuronal populations [5], including those from other brain regions and other neurons of the striatum. There is also significant loss of cortical neurons, especially of pyramidal neurons in layers III, V, and VI, including those that project directly to the striatum [6], [7], [8], [9].

Neuroinflammation, characterized by reactive gliosis and inflammatory processes in the central nervous system (CNS), has been considered as a prominent sign in various neurodegenerative diseases including HD [10], [11], [12], [13]. The pathogenic Htt protein influences microglia to secrete pro-inflammatory cytokines IL-1β, IL-6, IL-8, TNFα, component 1 subcomponent q (C1q), and IL-1α to facilitate a shift of normal astrocytes toward an A1 phenotype in the brain [14], [15], [16]. Accordingly, proinflammatory cytokines were elevated both centrally (in striatum and CSF) and peripherally (in plasma) in HD patients [14, 17, 18]. Therefore downregulation of glial activation and associated neuroinflammation would be a therapeutic approach for HD.

Aspirin, one of the most frequently used medicines in medical practice, is available over the counter. Recently we have demonstrated that oral aspirin is capable of upregulating regulatory T cells (Tregs) in an animal model of multiple sclerosis (MS) [19, 20] and lowering plaque load and improving hippocampal plasticity in an animal model of Alzheimer’s disease (AD) [21], [22], [23], [24]. However, oral aspirin has some side effects. Even at the baby dose, oral aspirin can cause gastrointestinal symptoms, such as heartburn, upset stomach, or pain. Therefore, we decided to treat mice with low-dose of aspirin via nebulization and demonstrated that aspirin nebulization lowered glial inflammation, reduced huntingtin pathology to exhibit neuroprotection in N171-82Q mouse model of HD, underlining the possible importance of aspirin nebulization in the treatment of HD.

## Materials and methods

### Reagents

Acetylsalicylic acid (aspirin), and all molecular biology–grade chemicals were purchased from Sigma-Aldrich (St. Louis, MO). Huntingtin and Iba1 antibodies were purchased from Abcam (Cambridge, MA). While GFAP antibody was procured from DAKO, inducible nitric oxide synthase (iNOS) antibody was purchased from BD Bioscience (San Jose, CA).

### Animals

Adult N171–82Q mice (B6C3-Tg(HD82Gln)81Gschi/J) were purchased from Jackson Laboratories. Experimental mice were housed under standard conditions with access to food and water *ad libitum*. Male N171–82Q mice were bred with female non-transgenic (nTg) B6C3 mice [25]. Mice positive for the mutated huntingtin gene were selected by genotyping. Animal maintenance and experiments were performed in accordance with the National Institutes of Health guidelines and were approved by the Institutional Animal Care and Use committee of the Rush University Medical Center (Chicago, IL).

### Nebulization of aspirin

Three-months-old N171-82Q transgenic (HD) mice were nebulized with 10 µg/mouse aspirin (solubilized in a volume of 100 μL saline) once daily for 5 min using the Buxco Inhalation Tower All-In-One Controller. A whole-body-chamber was fitted with aeroneb ultrasonic nebulizers supplied with air from a Buxco bias flow pump.

### Open field test

Open field test was performed to monitor the locomotor abilities of the animals on a horizontal plane [26, 27]. Movement associated parameters were captured with a camera linked to Noldus system and EthoVision XT software (Netherlands). The instrument records the overall movement abilities of the animals such as total distance moved, velocity, moving time, resting time, center time, and frequencies of movement. Before recording the movement, all experimental mice were placed inside the open field arena for 10 min daily for 2 consecutive days for training. Next day, animals were given rest and the following day each mouse was gently placed in the middle of the open field arena. After releasing the animal, data acquisition was started by the software for the next 5 min.

### Rotarod

Animals were placed on the rotating road against the direction of rotation [28, 29]. The machine was set to run at a gradual increasing speed of 4–40 rpm. The time spent on the rotating rod was recorded and the experiment ended once the animal slips from the rod to the base of the instrument.

### Grip test

Grip test was performed to measure the muscle strength of fore and hind limbs of the animals [25]. The test was conducted using a square platform made of metal wires. Mouse was placed on the middle of the metal platform and then the whole platform was reversed allowing the animal to hang from that platform by clasping. Animals were initially trained for two days and then after a gap of one day the experiment was performed. Time taken by each mouse to fall from the metal platform was recorded.

### Gait analysis

Mice were acclimatized by making them walk on a slanting platform for consecutive two days [29, 30]. Each mouse was given five trials each day to walk on the platform to the ascending direction. After a gap of one day, the experiment was performed. The gangway was covered with a long white paper and the limbs of the animals were painted with non-toxic black colored ink to get the impression of the footprints of each animal. Following the experiment, based on the footprints different gait parameters such as stride length, stride width, foot length and toe spread were measured. If any animal stopped or started walking in reverse direction, experiment for that animal was repeated [31].

### Pole test

The motor coordination was tested using a pole test [32, 33]. A vertical wood pole which was 50 cm in height and 1 cm in diameter was placed in the home cage. Mice placed head-up on top of the pole, orient themselves downward and descended the pole back into the home cage. The head of the mice was directed toward the top of the pole, and then measured the total time to climb down to the home cage. The experiment was measured for 1 min, and it was performed five times in total, and at least 5 min of rest was given between each experiment. The results of this study were used as the averages of the three fastest descending times. The pole test was performed by five per each group [32].

### Western blotting

It was performed as described [34, 35]. Briefly, the striatum and motor cortex region was isolated from mouse brain and homogenized in RIPA buffer containing 50 mM Tris-HCl, 1 mM EDTA sodium salt, 150 mM NaCl, 1 % Nonidet P-40, 0.5 % sodium deoxycholate, and protease inhibitor cocktail. Tissue homogenate was centrifuged at 17,500×*g* for 10 min at 4 °C, the resulting supernatant was collected, protein concentration was measured by Lowry method and samples for Western blotting were prepared. Protein samples were run in 8 % or 10 % SDS-PAGE followed by transfer to the nitrocellulose membrane. The membrane was probed with primary antibodies overnight at 4 °C. Next day, infrared fluorophore-tagged secondary antibodies (1:10,000; Jackson Immuno-Research) were added. Blots were scanned with an Odyssey infrared scanner (Li-COR, Lincoln, NE). Band intensities were quantified using ImageJ software (NIH, USA).

### Immunostaining

Immunohistochemistry was performed as described earlier [36, 37]. Mice were perfused transcardially with 4 % paraformaldehyde and the brains kept in 30 % sucrose solution at 4 °C. Coronal sections (40 μm thickness) were cut from the forebrain containing striatum and motor cortex. Sections were blocked with 3 % normal horse serum and 2 % BSA made in PBST containing 0.5 % Triton X-100 (Sigma-Aldrich) for 1 h. Then the sections were kept in primary antibodies and incubated at 4 °C temperature overnight under shaking conditions. Next day, the samples were washed with PBST at least three times, 10 min each, and further incubated with Cy2- or Cy5-labeled secondary antibodies (all 1:500; Jackson Immuno-Research) for 1 h under similar shaking conditions. Following several washes with PBST, sections were incubated for 5 min with 4′,6-diamidino-2-phenylindole (DAPI, 1:10,000; Sigma-Aldrich) for immunofluorescence. Whereas for immunohistochemistry, samples were kept in solution containing biotin-tagged secondary antibodies for 1 h followed by incubation in Vectastain A and B (Jackson Immuno-Research) mixture solution at room temperature. Sections were developed by 3,3′-diaminodenzidine (DAB; Sigma-Aldrich) solution containing peroxide. The sections were run in an ethanol and xylene (Fisher) gradient, mounted, and observed under confocal microscope (Zeiss). Mean fluorescence intensity (MFI) was measured using ImageJ and relative optical density of Htt staining was conducted using Fiji (ImageJ2) [33].

### Statistics

Statistics were performed using GraphPad Prism 10.1.1 (323). One-way ANOVA followed by Tukey’s multiple comparison test was performed for analyzing statistical significance among multiple samples, whereas unpaired two tailed t-test was performed to compare two samples. Values are expressed as mean ± S.E.M. The criterion for statistical significance was p<0.05.

## Results

### Aspirin nebulization reduces microglial activation in striatum and motor cortex of HD mice

**Figure 1:**
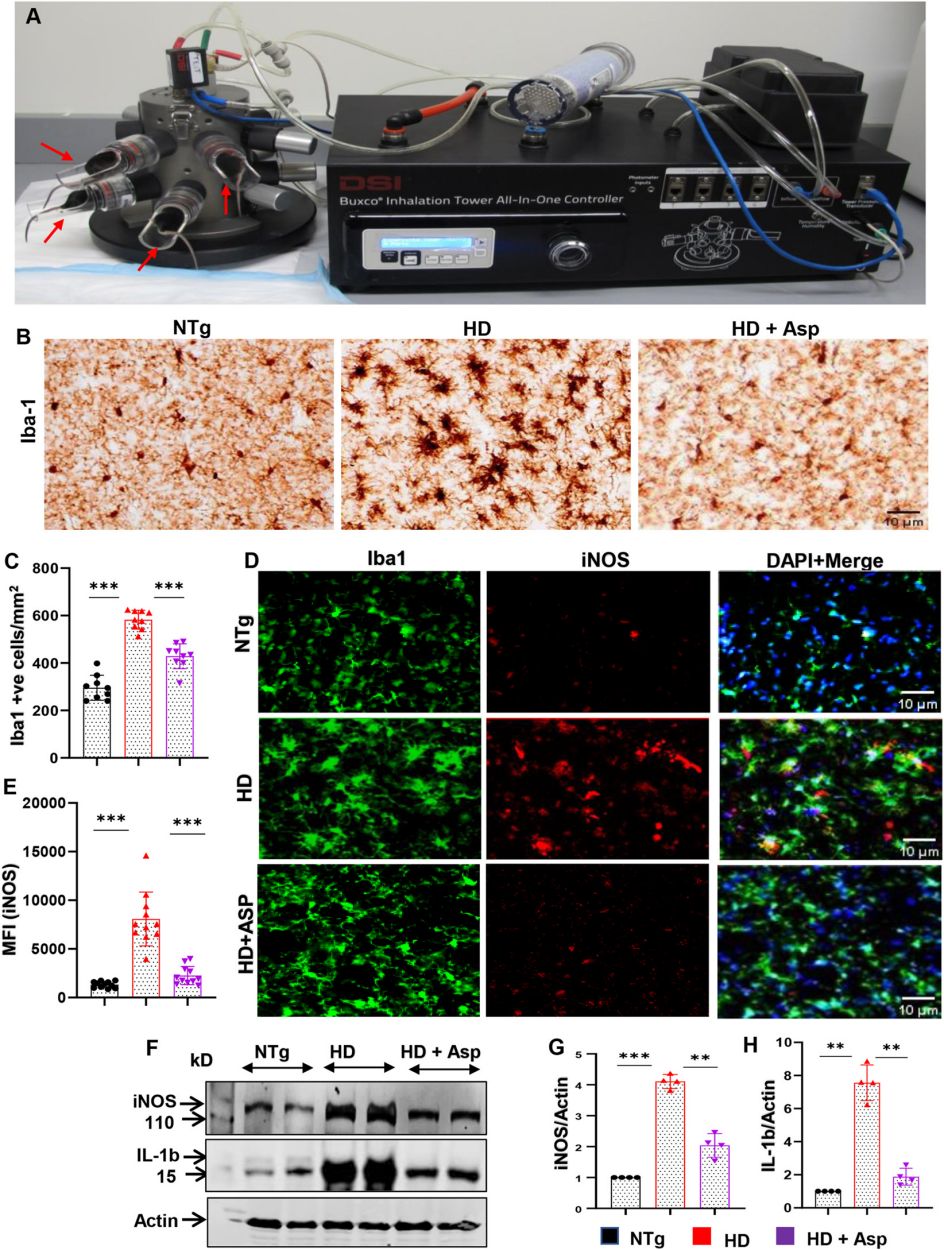
Nebulization of low-dose of aspirin inhibits glial activation and inflammation in the striatum of HD mice. (A) Buxco Inhalation Tower All-In-One Controller was used for nebulization. A whole body chamber was fitted with aeroneb ultrasonic nebulizers supplied with air from a Buxco bias flow pump. Three-months-old N171-82Q transgenic (HD) mice (n=5) were allowed to nebulize 10 µg/mouse aspirin (solubilized in a volume of 100 μL saline) once daily for 30 days. Red arrows indicated placement of mice in the nebulizer. (B) Microglial activation was monitored in stratal sections by DAB staining of microglial marker Iba1 followed by counting of Iba1-positive cells (C). Striatal sections were also double-labeled with antibodies against Iba1 and iNOS (D) followed by quantification of mean fluorescent intensity (MFI) of iNOS in microglia by image J (E). Two sections from each 5 mice per group were analyzed for counting and MFI. Protein expression of iNOS and IL-1β from striatum was evaluated by western blot analysis (F), and the ratio of band intensities of iNOS (G) and IL-1β (H) was calculated with respect to actin (loading control). One-way ANOVA followed by Tukey’s multiple comparison tests was performed for statistical analyses. ^**^p<0.01 and ^***^p<0.001. Data are represented as mean ± SEM (n=4 for immunoblotting and n=5 for staining).

Neuroinflammation is a normal and necessary process. However, chronic neuroinflammation is toxic for the CNS, leading to the pathogenesis of neuroinflammatory and neurodegenerative disorders [38], [39], [40]. Neuroinflammation in the HD brain is characterized by a reactive morphology of glial cells, including both microglia and astrocytes, along with the presence of inflammatory mediators in the brain parenchyma [25, 41, 42]. Aspirin (acetylsalicylic acid) is the most widely used nonsteroidal anti-inflammatory drug. Previously we examined its immunomodulatory effect in experimental autoimmune encephalomyelitis (EAE), an animal model of MS, and showed that oral administration of aspirin suppressed the clinical symptoms of EAE, reduced the infiltration of mononuclear cells, inflammation, and demyelination in the CNS [19]. In another study, oral aspirin also decreased cerebral plaque load and protected cognitive functions in an animal model of AD [21, 22]. Although oral administration represents the most convenient way of drug delivery, oral aspirin even at baby dose causes gastrointestinal symptoms, such as heartburn, stomach upset, or pain [43, 44]. Therefore, while investigating a way to bypass the direct exposure of aspirin to stomach and associated gastrointestinal tract, yet retaining its protective efficacies in the brain, we decided to treat mice via nebulization with a dose of aspirin that is much lower than the baby dose. Baby dose aspirin represents 81 mg aspirin/adult/day, which is roughly equivalent to 1.15 mg/kg body wt/d considering the average body weight of an adult human being as 70 kg. Therefore, N171-82Q transgenic mice were treated with 10 µg/mouse (equivalent to 0.4 mg/kg body wt considering the average body weight of a mouse as 25 g) once daily via nebulization (Figure 1A). This dose of aspirin is less than half of the baby dose. Since N171-82Q mice having an N-terminal fragment of Htt incorporating both exon 1 and exon 2 of the *Htt* gene with 82 polyglutamine, exhibit behavioral symptoms and protein aggregates at the age of 4 months with a life expectancy of 5–6 months, in this study, we started aspirin nebulization in N171-82Q (HD) mice from the age of 3 months when the animals showed some HD symptoms including loss of coordination and tremors.

**Figure 2:**
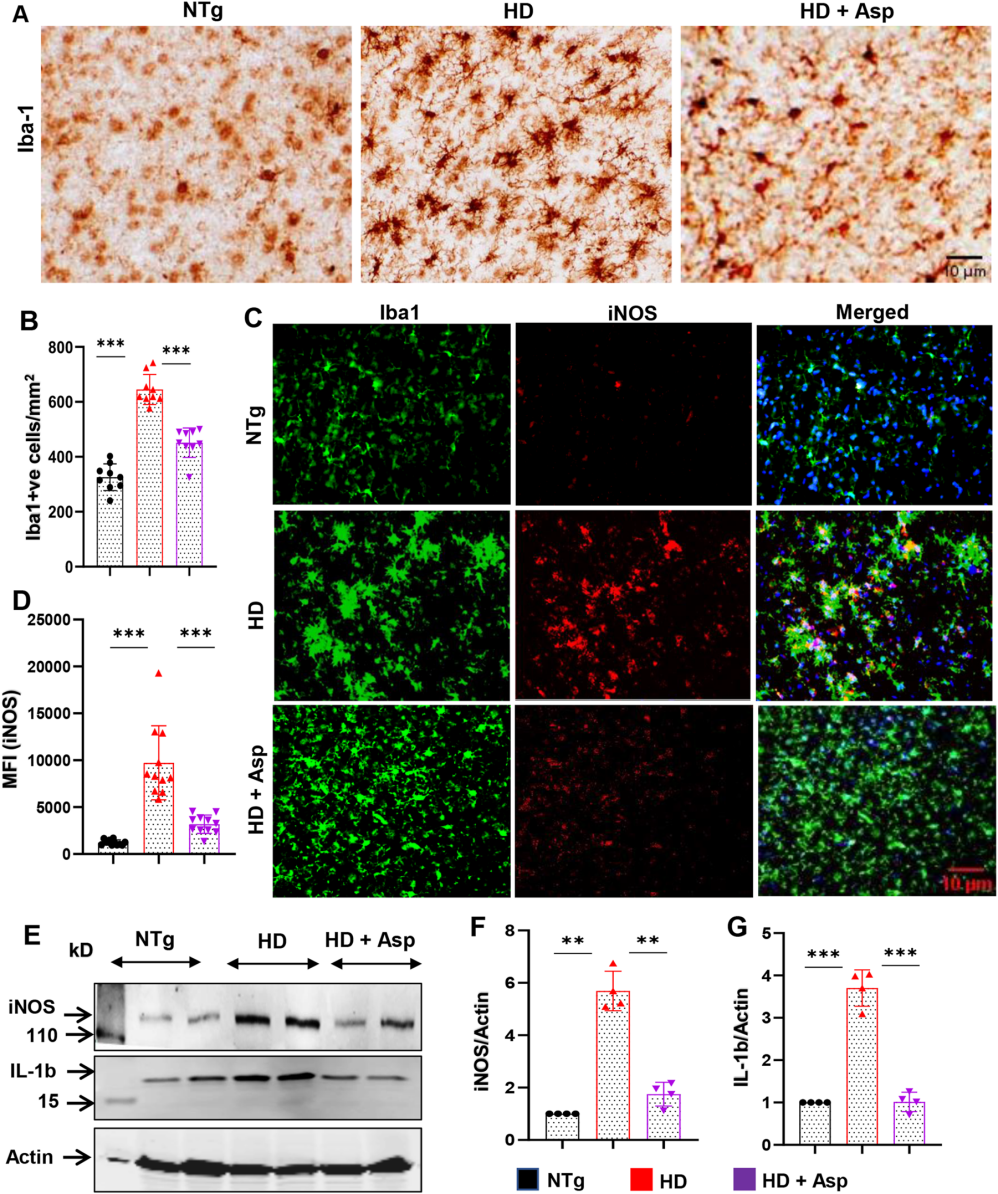
Nebulization of low-dose of aspirin inhibits glial inflammation in the motor cortex of HD mice. Three-months-old N171-82Q transgenic (HD) mice (n=5) were allowed to nebulize 10 µg/mouse aspirin (solubilized in a volume of 100 μL saline) once daily. After 30 d of nebulization, microglial activation was monitored in the motor cortex by DAB staining of microglial marker Iba1 (A) followed by counting of Iba1-positive cells (B). Sections were also double-labeled with antibodies against Iba1 and iNOS (C) followed by quantification of MFI of iNOS in microglia by image J (D). Two sections from each 5 mice per group were analyzed for counting and MFI. Protein expression of iNOS and IL-1β from the motor cortex was evaluated by western blot analysis (E). Actin was run as a loading control. The ratio of band intensities of iNOS (F) and IL-1β (G) was calculated with respect to actin. One-way ANOVA followed by Tukey’s multiple comparison tests was performed for statistical analyses. ^**^p<0.01 and ^***^p<0.001. Data are represented as mean ± SEM (n=4 for immunoblotting and n=5 for staining).

To examine the effect of aspirin nebulization on microgliosis, brain sections were DAB-stained with antibodies against microglial Iba1. As expected, we observed significant upregulation of Iba1 immunoreactivity in both striatum (Figure 1B and C) and motor cortex (Figure 2A and B) of HD mice as compared to non-Tg mice. However, nebulization of HD mice with low-dose aspirin for 30 d led to significant inhibition of Iba1 in both striatum (Figure 1B and C) and motor cortex (Figure 2A and B). It is known that upon activation, microglia are capable of releasing a number of proinflammatory molecules such as redox molecules (e.g. NO, inducible nitric oxide synthase or iNOS, superoxide ion, etc.), cytokines (e.g. IL-1β, IL-6, IL-8, TNF-α, etc.) [27, 45], [46], [47]. Therefore, to assess inflammation in the brain, double immunofluorescence staining was performed for nitrosative inflammatory marker, iNOS in Iba1-positive microglia. The results showed significant up-regulation of iNOS expression in both striatum (Figure 1D and E) and cortex (Figure 2C and D) of HD mice as compared to non-Tg mice, indicating increase in neuroinflammation in HD mice. On the other hand, aspirin nebulization significantly decreased microglial number and expression of inflammatory marker iNOS in both striatum (Figure 1D and E) and motor cortex (Figure 2C and D) of HD mice. To confirm this finding further, we performed Western blot analysis and found that the levels of iNOS and IL-1β were higher in striatum (Figure 1F–H) and motor cortex (Figure 2E–G) of HD mice in comparison to non-Tg mice and that aspirin nebulization was capable of suppressing the protein levels of both iNOS and IL-1β in striatum (Figure 1F–H) and motor cortex (Figure 2E–G) of HD mice. Therefore, microglial inflammation *in vivo* in the CNS of HD mice could be inhibited by nebulization of low-dose aspirin.

### Aspirin nebulization decreases astroglial activation in striatum and motor cortex of HD mice

**Figure 3:**
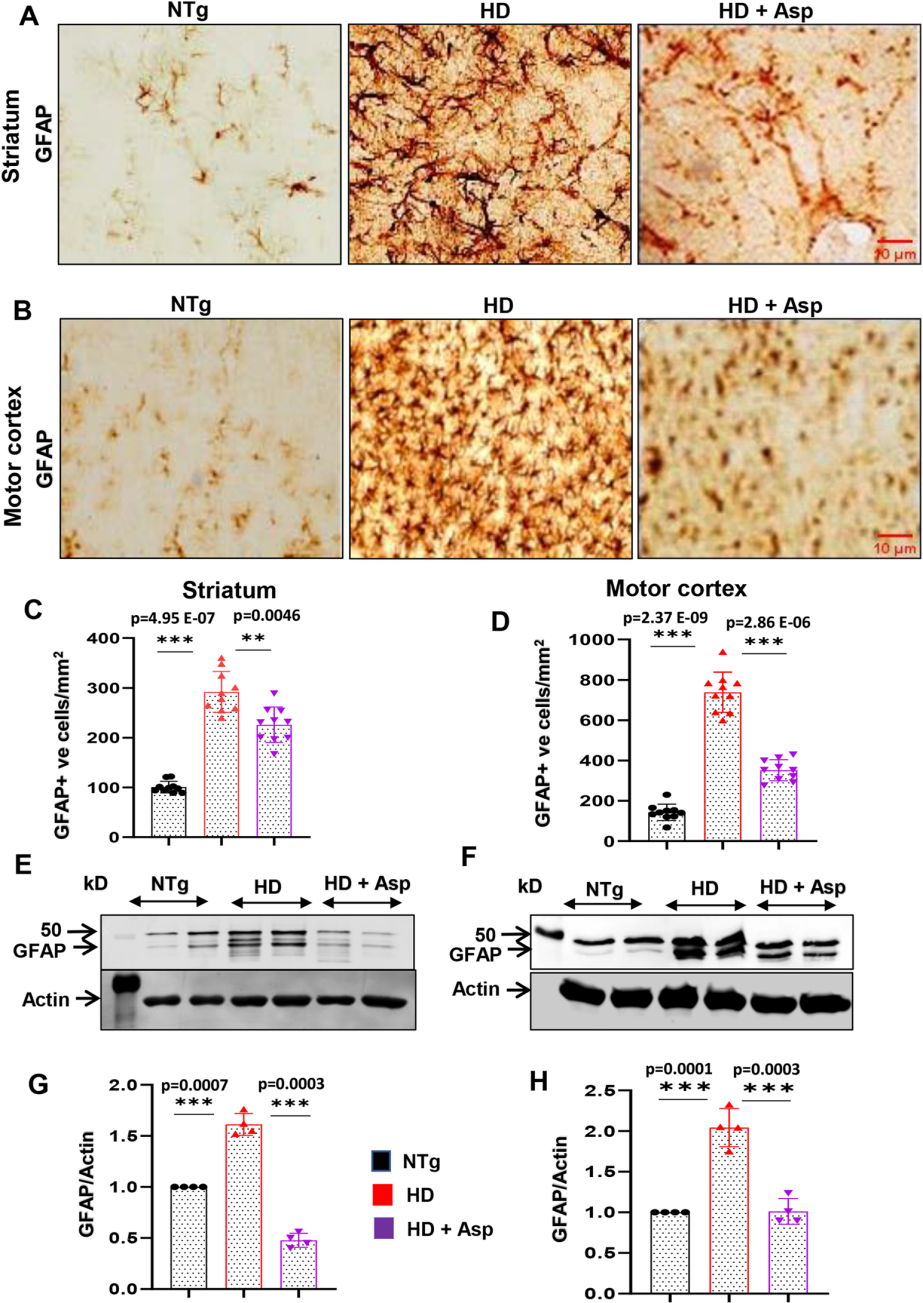
Aspirin nebulization attenuates astrogliosis in the striatum and motor cortex of HD mice. Three-months-old N171-82Q transgenic (HD) mice (n=5) were allowed to nebulize 10 µg/mouse aspirin (solubilized in a volume of 100 μL saline) once daily. After 30 d of nebulization, astroglial activation was monitored in the striatum (A) and motor cortex (B) by DAB staining of astroglial marker GFAP followed by counting of GFAP-positive cells in the striatum (C) and motor cortex (D). Two sections from each 5 mice per group were analyzed for counting. Protein expression of GFAP from the striatum (E) and motor cortex (F) was evaluated by western blot analysis. Actin was run as a loading control. The ratio of band intensities of GFAP (G, striatum; H, motor cortex) was calculated with respect to actin. One-way ANOVA followed by Tukey’s multiple comparison tests was performed for statistical analyses. ^**^p<0.01 and ^***^p<0.001. Data are represented as mean ± SEM (n=4 for immunoblotting and n=5 for staining).

Similar to microglial activation, astroglial activation also participates in the pathogenesis of neurodegenerative disorders [48], [49], [50]. Reactive astrocytes have been reported to be accumulated in proximity to degenerated neurons in HD brain [51] that are characterized by increased proliferation and astroglia marker glial fibrillary acidic protein (GFAP). Mounting evidence also suggests the presence of reactive astrocytes in pre-symptomatic HD carriers [52]. As evidenced by immunostaining with astrocytic marker GFAP, marked up-regulation of astrogliosis was observed in the striatum (Figure 3A) and motor cortex (Figure 3B) of HD mice as compared to nTg mice. It was corroborated by counting of GFAP-positive cells in the striatum (Figure 3C) and motor cortex (Figure 3D). Similarly, protein expression of GFAP was also elevated in the striatum (Figure 3E and G) and motor cortex (Figure 3F and H) of HD mice as balanced from nTg mice. However, similar to that found in Iba1-positive microglia, aspirin nebulization was also capable of reducing the number of GFAP positive cells (Figure 3A–D) and the expression of GFAP protein (Figure 3E–H) in the striatum and motor cortex of HD mice. These results suggest that aspirin nebulization is capable of reducing astroglial activation *in vivo* in the CNS of HD mice.

### Nebulization of low-dose aspirin decreases huntingtin (Htt) pathology in the brain and attenuates striatal atrophy

**Figure 4:**
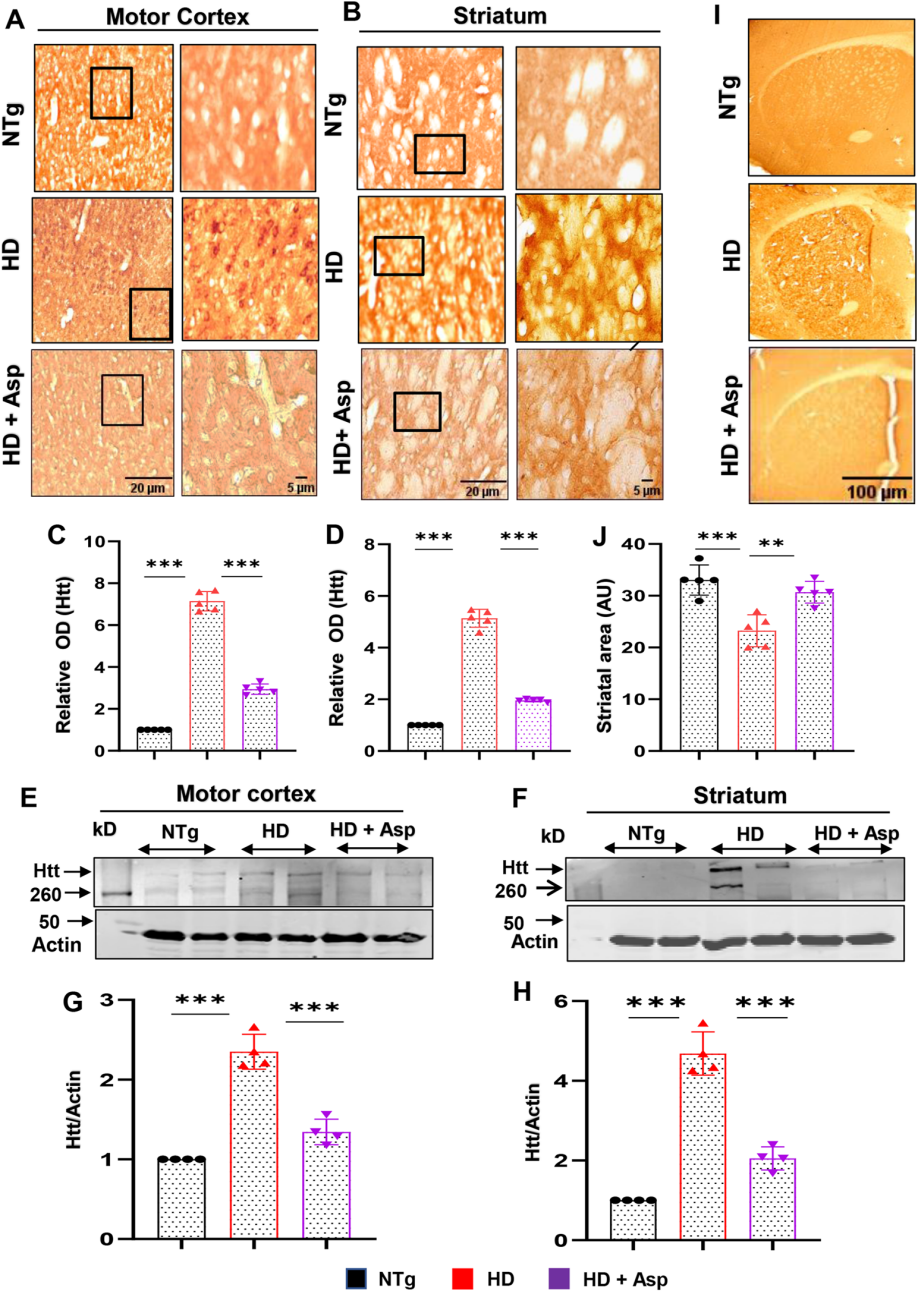
Aspirin nebulization decreases the level of mutant huntingtin and retains neuronal integrity in the brain of HD mice. Three-months-old N171-82Q transgenic (HD) mice (n=5) were allowed to nebulize 10 µg/mouse aspirin (solubilized in a volume of 100 μL saline) once daily. After 30 d of nebulization, Htt level was monitored in motor cortex (A) and striatum (B) by immunohistochemistry followed by measurement of optical density (O.D.) of Htt using Fiji, which was expressed as fold change with respect to non-transgenic (nTg) control (C, motor cortex; D, striatum). Scale bars for lower (20X) and higher (60X) magnification images were kept as 20 and 5 µm, respectively. Protein level of Htt from the motor cortex (E) and striatum (F) was evaluated by western blot analysis. Actin was run as a loading control. The ratio of band intensities of Htt (G, motor cortex; H, striatum) was calculated with respect to actin. Striatal area was measured using ImageJ and the average area is shown as arbitrary units (AU). One brain section from each of 5 mice per group were considered for the analysis (I, J). One-way ANOVA followed by Tukey’s multiple comparison tests was performed for statistical analyses. ^**^p<0.01 and ^***^p<0.001. Data are represented as mean ± SEM (n=4 for immunoblotting and n=5 for staining).

Since aspirin nebulization reduced microglial and astroglial activation *in vivo* in the brain of HD mice, next, we investigated whether the same treatment had any effect on Htt pathology. As expected, the immunostaining results showed marked deposition of Htt in neurons of motor cortex (Figure 4A) and striatum (Figure 4B) of HD mice as compared to nTg mice. This finding was confirmed by quantification of Htt optical density in the motor cortex (Figure 4C) and striatum (Figure 4D). However, neuronal Htt aggregation significantly reduced in the motor cortex (Figure 4A and C) and striatum (Figure 4B and D) of HD mice after aspirin treatment. To confirm this finding further, we performed Western blot analysis of Htt protein, which also showed a significant decrease in aggregated Htt in the motor cortex (Figure 4E and G) and striatum (Figure 4F and H) of HD mice after aspirin nebulization.

Brain atrophy or cerebral atrophy is a condition in which the brain or a particular region of the brain decreases or shrinks in size. Several studies have reported atrophy of variable severity in the brains of patients with HD [53, 54]. Accordingly, HD mouse brains also showed loss of striatal area in comparison to age-matched nTg brains (Figure 4I and J). However, consistent to the attenuation of glial activation and reduction of Htt pathology, aspirin nebulization of HD mice led to significant reduction in the loss of striatal area (Figure 4I and J). Therefore, aspirin nebulization is capable of preventing striatal atrophy in HD mice.

### Aspirin nebulization improved general motor behavior of HD mice

**Figure 5:**
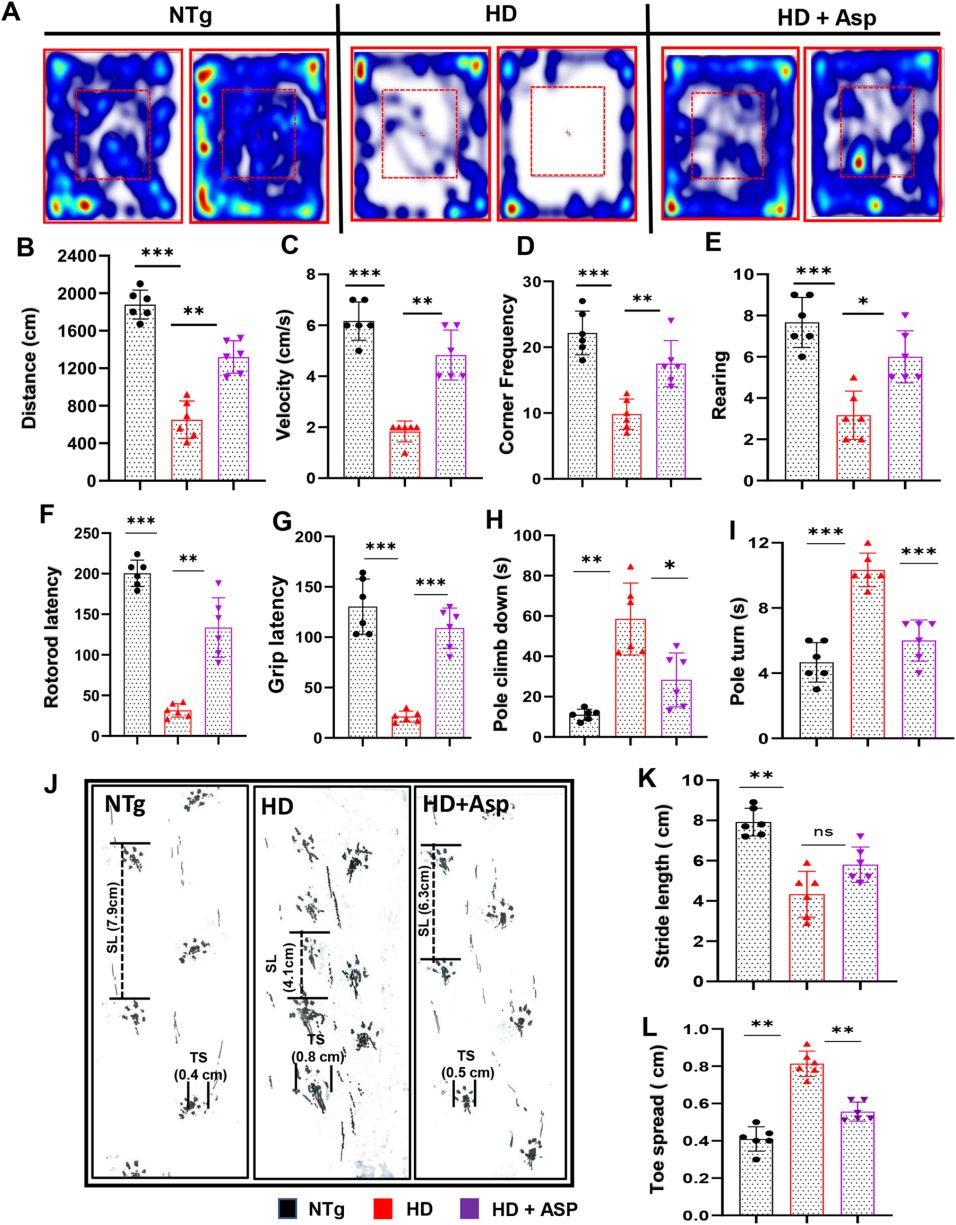
Nebulization of low-dose aspirin diminishes motor behavioral impairment of HD mice. Three-months-old N171-82Q transgenic (HD) mice (n=6) were allowed to nebulize 10 µg/mouse aspirin (solubilized in a volume of 100 μL saline) once daily. After 30 d of nebulization, general locomotor activity was analyzed by open field test (A, representative heat map; B, total distance traveled; C, velocity; D, corner frequency; E, rearing). Feet movement of animals was evaluated by rotarod test (F), whereas muscle strength was assessed by grip test (G). Motor co-ordination in mice was performed by pole test (H, I), and footprints (J). Stride length (J, K) and toe spread (J, L) of mice obtained from the gait analysis were calculated manually. One-way ANOVA followed by Tukey’s multiple comparison tests was performed for statistical analyses. ^*^p<0.05; ^**^p<0.01; ^***^p<0.001; ns, not significant. Data are represented as mean ± SEM of 6 mice per group.

Since HD is characterized by progressive motor dysfunction, we checked whether nebulization of aspirin could improve functional impairments in HD mice. Therefore, we monitored general locomotor activity of HD mice by the open field test. A video camera 6 *(Basler Gen I Cam – Basler acA 1300–60)* connected to a Noldus computer system was fixed on top facing-down on the open-field arena for recording general locomotor activity. Representative heat maps summarizing the overall activity of mice on the open-field apparatus are shown in Figure 5A. Open-field behavioral analysis indicated a significant improvement in movement parameters such as distance travelled (Figure 5B), velocity (Figure 5C), corner frequency (Figure 5D), and rearing (Figure 5E) in aspirin treated HD mice as compared to untreated HD mice. In fact, some of these parameters of aspirin-treated HD mice were almost comparable to that of nTg mice, indicating significant recovery of locomotor activities close to control level by aspirin treatment. We also performed rotarod test, for motor coordination (Figure 5F), and grip test for muscle strength (Figure 5G). Results showed higher latency time taken by aspirin treated HD mice in rotarod and grip test compared to non-treated HD mice (Figure 5F and G). The pole test was performed to assess basal ganglia related movement disorders in our experimental HD mice, which clearly showed that aspirin treated HD mice took less time to climb down the pole (Figure 5H) and for pole turn (Figure 5I) as compared to untreated HD mice. Next, we conducted footprint analysis for measuring grit (Figure 5J). While the stride length of HD animals was significantly lower than the nTg animals (Figure 5J and K), the toe spread increased in HD mice as compared to nTg mice (Figure 5J and L). However, aspirin-nebulized HD mice performed better than untreated HD mice on footprint test as evident from stride length (Figure 5J and K) and toe spread (Figure 5J and L). Together, these results suggest that aspirin nebulization improves general motor behavior of HD mice.

## Discussion

Huntington’s disease (HD) is a devastating neurodegenerative disorder caused by abnormal trinucleotide CAG repeats and a progressive degeneration of neurons in basal ganglia and brain cortex, leading to motor, cognitive, and psychiatric symptoms. There is no disease modifying therapy for HD and the current treatment is aimed at managing chorea and psychiatric symptoms. Therefore, it is important to find out an effective drug for HD and during the search for effective therapeutics, several drugs have been tested for clinical trials, including immunomodulators, which failed to meet the efficacy endpoints [55]. Here, we demonstrate the first evidence that aspirin, one of the most frequently used pharmaceutics in medical practice, significantly reduces HD pathology in a mouse model. The main highlights of this study are: 1. Reduction of gliosis and inflammation in the striatum and motor cortex of HD mouse brain by aspirin nebulization; 2. Down-regulation of Htt pathology in the striatum and motor cortex of HD mice by aspirin; 3. Prevention of brain atrophy in HD mice by aspirin; 4. Improvement of motor functions in HD mice by aspirin. These results indicate that aspirin may be beneficial for neuroprotection in HD.

The Htt pathology is a key neuropathological hallmark of HD and usually aggregated Htt moieties are detected as inclusion bodies either in the cytoplasm or the nuclei [56, 57]. The relative contribution of soluble and aggregated forms of Htt to the pathogenesis of HD is still unclear. Nevertheless, it is believed that the presence of N-terminal mHtt fragments could lead to HD pathogenesis [58]. Nuclear incorporation can be detected before symptom onset in presymptomatic gene carriers [59]. Degeneration in HD brains initially involves the dorsal striatum where medium spiny neurons present nuclear inclusions and dystrophic neuritis [10]. In the cerebral cortex, the number of cortical pyramidal neurons of HD patients is reduced [60]. The above observation has been well documented in human patients as well as many experimental animal models with the expression of different lengths of PolyQ in the mHtt [61, 62]. Here, it is nice to see the decrease in Htt pathology in both striatum and motor cortex of N171-82Q transgenic mice by aspirin treatment. Although here, we have not tested the mechanism behind aspirin-mediated decrease in Htt pathology, it is believed that loss of autophagy may contribute to HD pathogenesis and that upregulation of autophagy may play an important role in reducing the Htt pathology [63]. We have demonstrated that aspirin is capable of upregulating TFEB, increasing lysosomal biogenesis and stimulating autophagy in brain cells [21]. Therefore, by increasing lysosomal biogenesis and autophagy, aspirin may reduce cerebral Htt load.

Neuroinflammation, characterized by reactive gliosis and production of soluble inflammatory factors in the CNS [64], coupled with the generation of oxidative stress or nitrosative stress and neurotoxicity is another key feature of HD brains [65, 66]. However, the exact mechanisms by which neuroinflammation contributes to HD pathology are still unclear. It is also not known whether immune activation is a pathological mechanism of HD or the consequence of neuronal/glial disfunction. However, several studies suggest that reducing neuroinflammation may be beneficial for HD. Here, we have noticed significant reduction in microgliosis and astrogliosis and associated neuroinflammation in different parts of the brain of HD mice after aspirin treatment. Although we have not investigated mechanisms by which aspirin reduced the levels of different proinflammatory molecules in the brain of HD mice, promoter regions of proinflammatory molecules harbor the DNA binding site for NF-κB and the inhibition of NF-κB activation reduces the induction of proinflammatory molecules [12, 13, 67]. Aspirin is also known to inhibit the activation of NF-κB [68]. Therefore, by suppressing NF-κB activation, aspirin treatment may inhibit the level of proinflammatory molecules (e.g. iNOS, IL-1β, etc.) in the brain of HD mice. Moreover, NO has been shown to be directly involved in the upregulation of CD11b and microgliosis [69] as well as the increase in GFAP and associated astrogliosis [50], indicating that by suppressing iNOS/NO pathway, aspirin treatment may attenuate microgliosis and astrogliosis in the brain of HD mice.

In addition to the suppression of neuroinflammation, there are also other pathways by which aspirin may exhibit neuroprotection in HD mice. For example, brain-derived neurotrophic factor (BDNF) is a crucial regulator of neuronal growth, differentiation, and survival [70] and it has been reported that the selective striatal neurons degeneration may be caused by the depletion of brain-derived neurotrophic factor (BDNF) in HD [71]. Accordingly, reduced BDNF has been reported in post-mortem HD brain [72] and the concentration of BDNF is also low in the striatum of R6/1 and zQ175 mice compared to the wild type counterparts [73]. Since aspirin is capable of increasing BDNF in neurons and astrocytes [74], aspirin treatment may also exhibit neuroprotection in HD mice via upregulation of BDNF.

Aspirin is a widely-used over-the-counter medicine and an important aspect of this work is the delineation of a new mode of delivery of aspirin for neuroprotection in the CNS. Although recently, we have shown that oral administration of aspirin protects mice from EAE (an animal model of MS) [19], reduces plaques and upregulates hippocampal plasticity in an animal model of AD [22, 23] and increases tyrosine hydroxylase in the brain of A53T mouse model of PD [75], oral aspirin has some side effects. Even at the baby dose, aspirin elicits gastrointestinal symptoms, such as heartburn, upset stomach, or pain [43, 44]. Therefore, here, we used the nebulization technique to bypass the direct exposure of aspirin to stomach and reduce the chance of gastrointestinal problems. Moreover, by using the nebulization approach, we have established the neuroprotective efficacy of aspirin in N171-82Q mouse model of HD at a dose that is less than half of the baby dose. While the baby dose of aspirin is 81 mg/adult/day or 1.15 mg/kg body wt/d considering 70 kg as the average body weight of an adult human being, our nebulization dose of 10 µg/mouse/d aspirin is approximately equivalent to 0.4 mg/kg body wt/d considering the average body weight of a mouse as 25 g.

In summary, here, we demonstrate suppression of glial activation, reduction in huntingtin pathology and improvement in locomotor activities in a mouse model of HD by nebulized aspirin at a dose of 10 µg/mouse/d or 0.4 mg/kg body wt/d, which is roughly equivalent to 28 mg/adult/day. It is less likely that nebulization of aspirin at a dose less than half of the baby dose will cause any gastrointestinal problems.
